# Adamantinoma: An Uncommon Cause of Bone Pain in a Young Adolescent Male

**DOI:** 10.7759/cureus.50214

**Published:** 2023-12-09

**Authors:** Nikita Bora, Shivali V Kashikar, Pratap Parihar, Nishant Raj, Neha D Shetty, Bhagyasri Nunna

**Affiliations:** 1 Radiodiagnosis, Datta Meghe Institute of Higher Education and Research, Wardha, IND

**Keywords:** bone tumor, mri, tibia, radiology, adamantinoma

## Abstract

Adamantinoma, an uncommon low-grade primary malignant bone tumor, rarely causes leg pain in adolescents and typically manifests in the lower extremities, with a notable preference for the tibia, although occurrences in other bones such as the femur, fibula, and pelvis have been documented. Instances of local recurrence and regional metastasis are infrequent. This case report aims to comprehensively review the clinical presentation, imaging features, histological findings, and management of adamantinoma. The presented case involves a 17-year-old male patient with a four-year history of edema and discomfort in the right anterior leg. Radiographic examination of the proximal tibia revealed a well-defined, expansile lytic-sclerotic lesion with multiple septae and a partially sclerotic border. Subsequent magnetic resonance imaging (MRI) confirmed the nature of the lesion, and a biopsy, followed by histological analysis, confirmed the diagnosis of adamantinoma. This case highlights the significance of a multidisciplinary approach, emphasizing close collaboration among radiology, pathology, and orthopedic oncology in adamantinoma management. Long-term follow-up is imperative for monitoring recurrence and administering timely therapy. The objective of this case report is to contribute to an improved understanding of adamantinoma and offer guidance on the treatment of this uncommon bone tumor.

## Introduction

Adamantinoma is a rare malignant bone tumor primarily seen to occur in the tibia. It also tends to occur in other bones, such as the femur, ulna, humerus, and radius. The origin of this lesion is not very well known, but its name has been derived from its similarity to ameloblastoma found in the jaw. It has no gender preference; males and females are equally susceptible. However, it is predominantly seen to affect young adults [1]. The clinical course is slow and progressive, characterized by swelling, pain, and deformity. Its histogenesis is uncertain, but there is evidence of epithelial differentiation [2-4].

Adamantinoma accounts for a mere 0.1-0.5% of primary bone tumors [5]. In a few instances, cutaneous metastasis is also seen, albeit a local recurrence, and distant metastases may happen years after the initial lesion [6-9]. From a radiographic perspective, the adamantinoma appears as a bubbly, expansile, lytic-sclerotic lesion on the cortical diaphyseal lesion and is positioned eccentrically. Larger tumors may also exhibit involvement of soft tissues. Adamantinoma has many radiological and histological characteristics with osteofibrous dysplasia; nevertheless, because of its locally aggressive nature, it is crucial to differentiate it from benign disorders. Magnetic resonance imaging (MRI) is a valuable tool in this regard [9]. Histopathological analysis of adamantinoma development patterns reveals a variety of morphologies, including tubular, squamoid, spindle, and basaloid.

## Case presentation

A 17-year-old boy presented with a four-year history of pain and swelling in the right leg. The pain was insidious in onset, non-progressive, and dull aching. There was a history of trauma to the same side of the leg two years back while playing kabaddi, following which he had a fracture of the tibia. He was managed with cast application for the same for three months and became fine after cast removal.

On physical examination of the right leg, the anterior aspect of the tibial contour was mildly deformed with mild tenderness; however, there was no obvious redness, warmth, discharge, or other inflammatory signs over the site. The range of motion of the right knee was within normal limits, distal circulation was intact, and no distal neurovascular deficit was observed. There were no signs of any systemic involvement. His laboratory findings were unremarkable.

**Figure 1:**
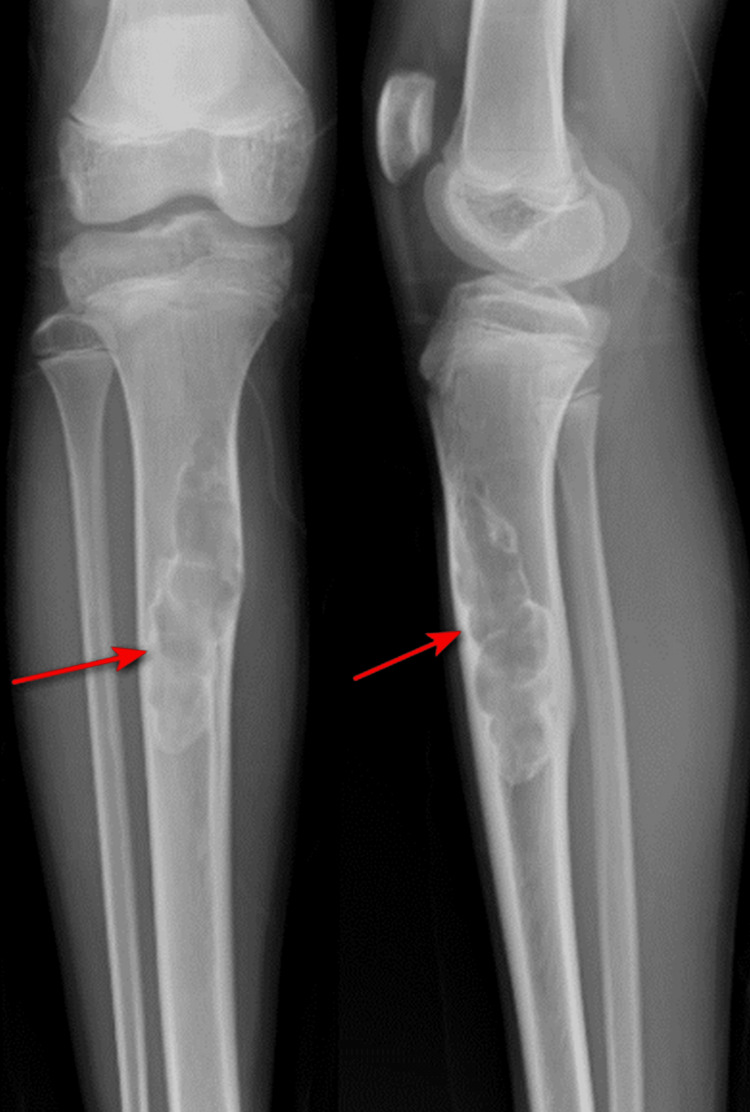
Anteroposterior and lateral plain radiographs of the right lower limb showed a well-defined, expansile lytic-sclerotic lesion (red arrow).

Anteroposterior and lateral plain radiographs (Figure 1) of the right lower limb showed a well-defined, expansile lytic-sclerotic lesion (red arrow) with a partially sclerotic border and multiple septa within the lesion in the proximal tibia. 

**Figure 2:**
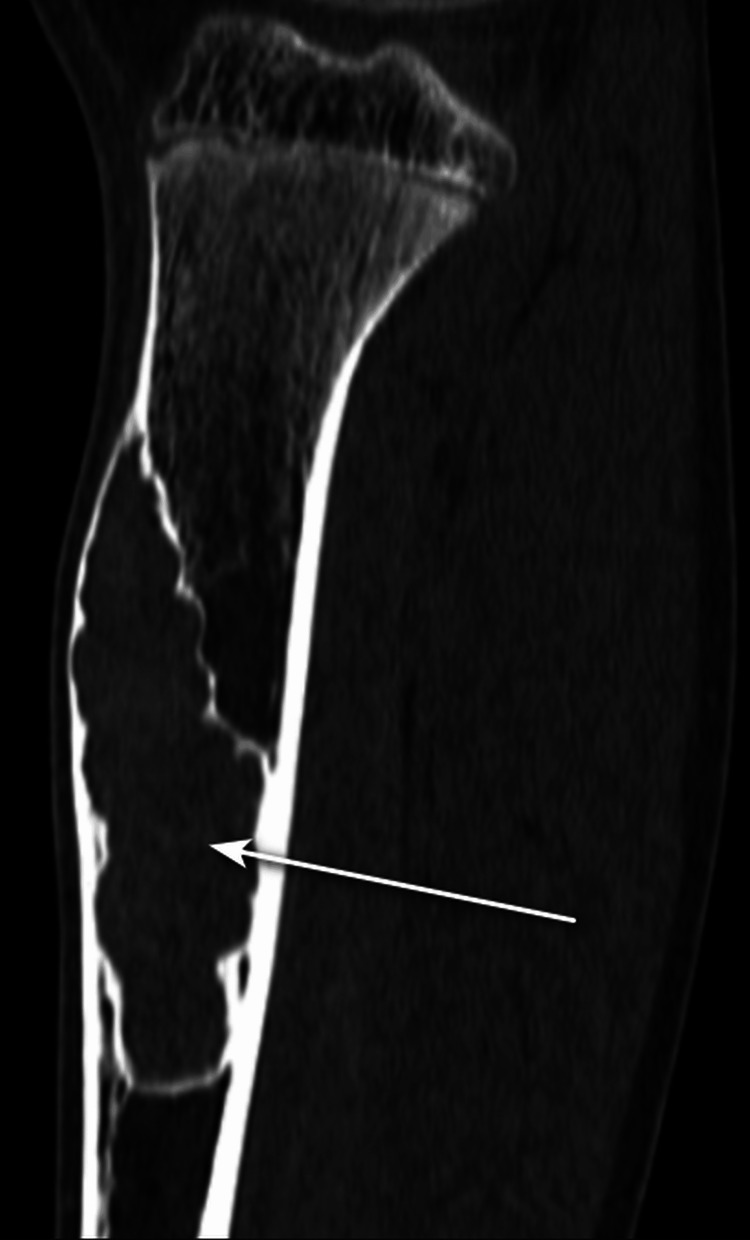
Sagittal CT section of the tibia in bone window showing expansile lytic lesion (white arrow). CT: computed tomography

On the computed tomography (CT) scan (Figure 2), there was an expansile lytic lesion (white arrow) noted along the anterior aspect of the tibia involving the cortex and medulla. 

**Figure 3:**
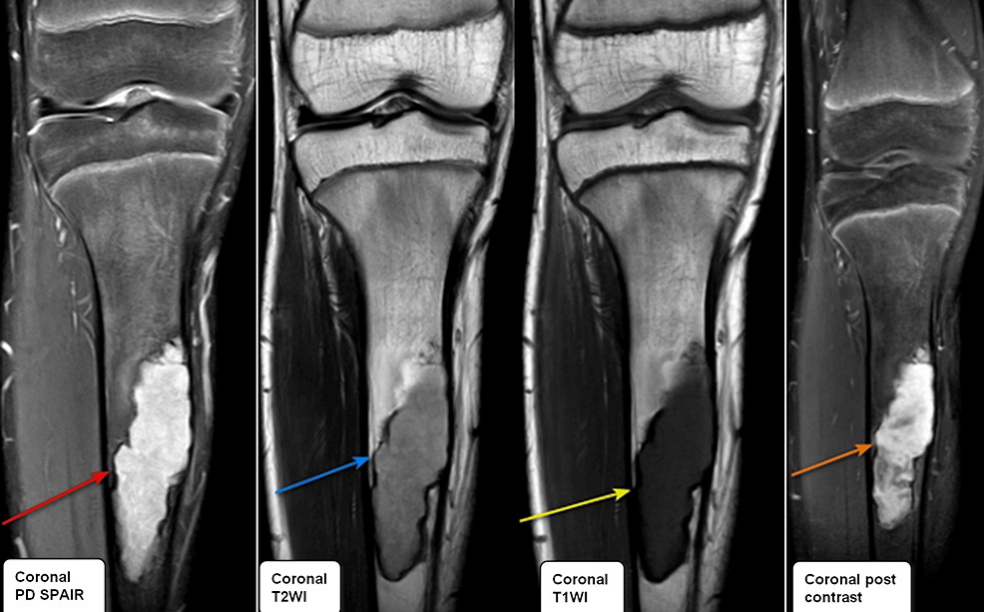
Coronal PD-SPAIR, coronal T2WI, coronal T1WI, and coronal post-contrast images (left to right) showing altered signal intensity lesion appearing hyperintense on PD-SPAIR (red arrow), showing intermediate signal on T2WI (blue arrow), appearing hypointense on T1WI (yellow arrow), and showing intense post-contrast enhancement (orange arrow). PD-SPAIR: proton density-spectral adiabatic inversion recovery, T2WI: T2-weighted imaging, T1WI: T1-weighted imaging

An MRI scan (Figure 3) was performed to assess the lesion further. On MRI, a defined cortical-based diaphyseal lesion was seen in the anterior aspect of the proximal tibial shaft. The lesion showed a narrow zone of transition, appearing hyperintense on proton density-spectral adiabatic inversion recovery (PD-SPAIR) (red arrow), isointense on T1-weighted imaging (T1WI) (yellow arrow), and mildly hyperintense on T2-weighted imaging (T2WI) (blue arrow) with vivid post-contrast enhancement (orange arrow). There was no evidence of periosteal reaction or any soft tissue involvement. A provisional diagnosis of adamantinoma was kept. 

**Figure 4:**
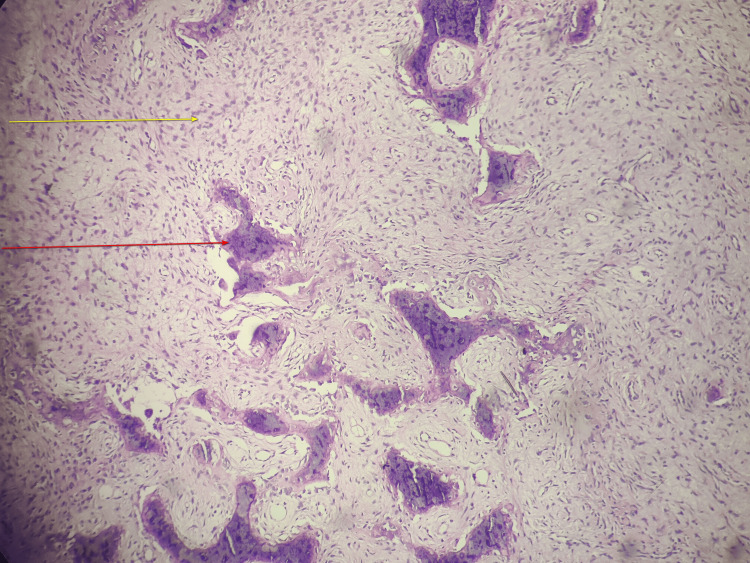
Hematoxylin and eosin-stained section showing a combination of epithelial and fibrous mesenchymal cells (yellow arrow) with epithelial cells arranged in palisading pattern (red arrow).

A biopsy of the lesion was performed, and histopathological examination (Figure 4) showed a combination of epithelial and fibrous mesenchymal cells (yellow arrow); the nest of epithelial cells was seen arranged in a palisading pattern (red arrow); the histopathological features confirmed the diagnosis of adamantinoma.

**Figure 5:**
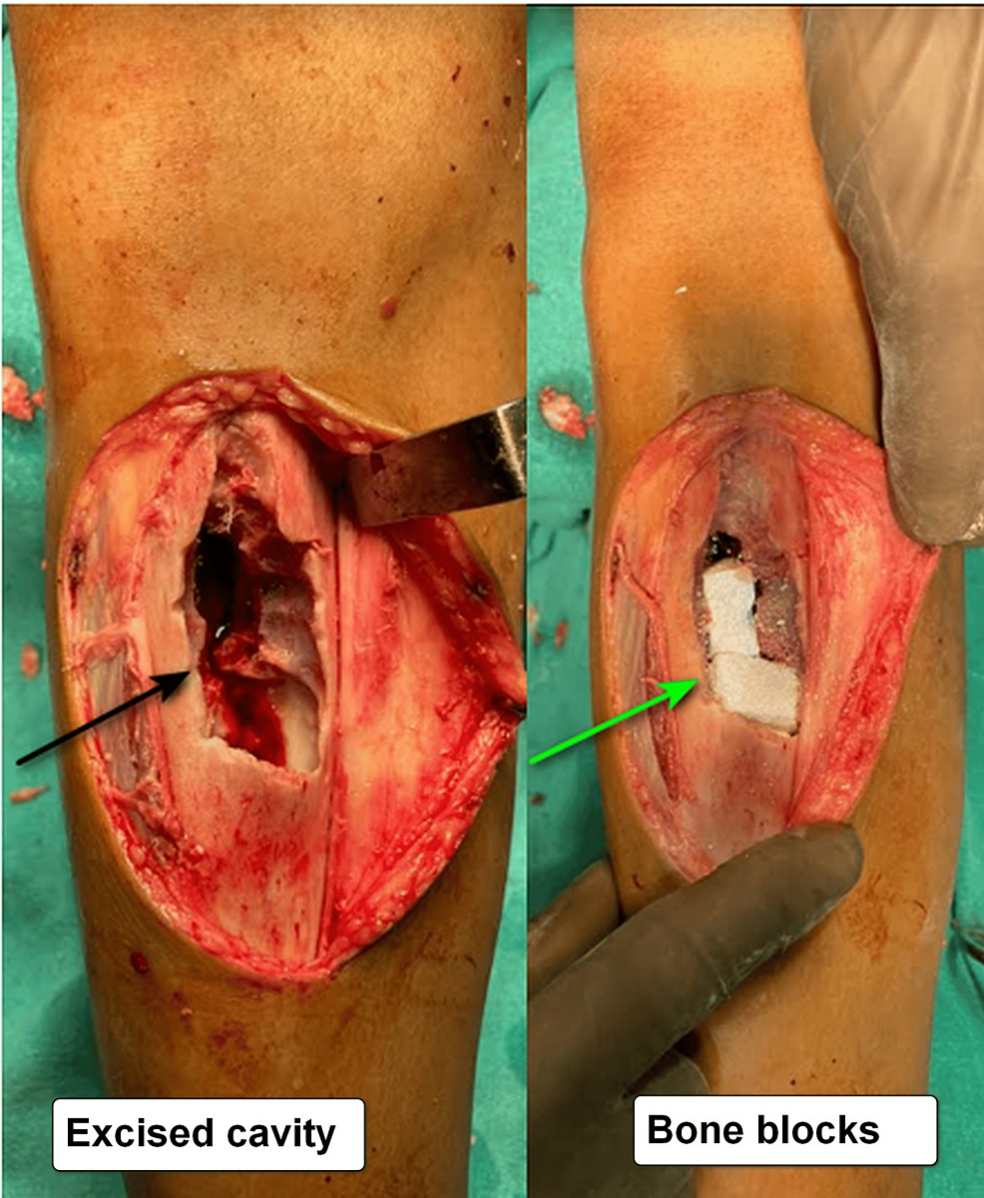
Postoperative image showing excision and curettage of the lesion (black arrow) with hydroxyapatite bone blocks filling the excised cavity (green arrow).

The patient was undertaken for excision and curettage (Figure 5) of the lesion (black arrow), following which the cavity in the anterior aspect of the tibia was plugged with hydroxyapatite bone blocks and sealed with bone wax (green arrow). Postoperative X-rays confirmed the adequate filling of the cavity with no evidence of breakage in the bone. 

Postoperative physiotherapy was started, and the patient had no postoperative complications. Suture removal was done on post-op day 12, and the patient was subsequently discharged and advised for regular follow-up.

## Discussion

Our case emphasizes the hallmark of interdisciplinary teamwork. In our case, we have discussed the possibility of an adolescent boy with long-term discomfort in the form of constant dull aching pain in the anterior aspect of his right leg, which became the pathological site for fracture after a trivial trauma while playing. With a multidisciplinary approach, including imaging on both conventional radiographs and 3T MRI sequences with additional post-contrast images followed by histopathological confirmation of the malignant, we had helped the onco-surgeons to make a good call. Following a year of monitoring, there was no recurrence of the lesion.

Adamantinoma is a rare, low-grade malignant bone tumor that occurs predominantly in the tibia of the lower extremities but can also be found in other bones, such as the femur, fibula, and pelvis [10]. It is considered a slow-growing tumor with a propensity for local recurrence and regional metastasis [11]. The diagnosis of adamantinoma can be challenging due to its rare occurrence and overlapping imaging and histological features with other benign and malignant bone tumors [12]. A multidisciplinary approach, including close collaboration between radiology, pathology, and orthopedic oncology, is crucial in managing this tumor [13]. The histological diagnosis of adamantinoma is based on identifying characteristic features, including adamantinoma-like stroma and squamous differentiation [11]. A wide surgical resection with negative margins is considered the mainstay of treatment for adamantinoma, and adjuvant radiotherapy may be considered in high-risk cases [13].

Khémiri et al. [6] described a case of a 50-year-old female who presented with a long history of pain in the mid part of the left leg without trauma. After imaging and workup, adamantinoma was identified as the cause. She underwent above-knee amputation as, in her case, the knee joint was involved with its vascular axes. After a year of surveillance, she presented with breathlessness and was diagnosed with pulmonary metastasis. Their study stresses the value of monitoring patients even after the original tumor has been completely removed.

Anoumou et al. [14] described a case of adamantinoma in a 41-year-old male patient who presented with tender swelling along the anterior aspect of the right leg. A plain radiograph showed a lytic lesion along the anterior part of tibia with involvement of adjacent soft tissue, and the patient subsequently underwent biopsy with immunohistochemistry study wherein epithelial and osteofibrous components were identified in varying proportions, the arrangement of epithelial cells in palisading pattern over a background of fibrous stroma being a key histological feature.

## Conclusions

This case report highlights the importance of an interdisciplinary team approach to managing adamantinoma, including close collaboration between the radiologist, histopathologist, and orthopedic oncologist team and long-term follow-up to monitor for recurrence and to provide timely response and work accordingly.
